# Comparative analysis of F-18 FDG PET/CT images between scrub typhus and systemic lupus erythematosus

**DOI:** 10.1038/s41598-024-65256-1

**Published:** 2024-07-03

**Authors:** Yeon-Hee Han, Joo-Hee Hwang, Yunjung Choi, Chang-Seop Lee

**Affiliations:** 1 Department of Nuclear Medicine, Cyclotron Research Center, Molecular Imaging and Therapeutic Medicine Research Center, Jeonbuk National University Medical School and Hospital, Jeonju, 54896 Republic of Korea; 2 Department of Internal Medicine, Jeonbuk National University Medical School and Hospital, Jeonju, 54896 Republic of Korea; 3 Division of Rheumatology, Department of Internal Medicine, Jeonbuk National University Medical School and Hospital, Jeonju, 54896 Republic of Korea; 4 Research Institute of Clinical Medicine of Jeonbuk National University-Biomedical Research Institute of Jeonbuk National University Hospital, Jeonju, 54907 Republic of Korea

**Keywords:** Rheumatic diseases, Bacterial infection

## Abstract

This study evaluated the use of F-18 fluorodeoxyglucose (FDG) PET/CT imaging to differentiate between scrub typhus and systemic lupus erythematosus (SLE) in patients presenting with lymphadenopathy. We carried out a retrospective analysis of 18 scrub typhus patients and seven SLE patients, using various imaging parameters, including lymph node size, spleen and liver lengths, the distance between the two farthest lesions (D_max_), and assessments of glucose metabolism. On FDG PET images, we measured the maximum standardized uptake value (SUV_max_) of the lymph nodes, spleen, and liver and the mean standardized uptake value (SUV_mean_) of the liver and spleen. The D_max_ values of scrub typhus patients were significantly longer than those of SLE patients, indicating that lymphadenopathy is more generalized in the patients with scrub typhus. The SUV_max_ values for the lymph node, spleen, and liver were also higher in patients with scrub typhus, while the SUV_mean_ of the liver and spleen did not differ between the two groups. This study is the first to compare FDG PET/CT images between these two conditions, suggesting the potential of this imaging modality to provide critical diagnostic distinctions.

## Introduction

The etiologies of lymphadenopathy (LAP) encompass a wide variety of conditions, such as infection, malignancy, autoimmune diseases, and certain medications^1,2^. Differentiating among these conditions is a significant clinical challenge, with multiple studies confirmed the difficulty in distinguishing benign LAPs from malignant ones, such as sarcoidosis versus lymphoma^3–6^.

Imaging techniques, particularly fluorine-18 (F-18) fluorodeoxyglucose (FDG) positron emission tomography/computed tomography (PET/CT), play a vital role in diagnosing different LAPs^7–11^. Although developed for use in oncology, FDG PET/CT, is now used for infectious and inflammatory diseases. Patients with acute scrub typhus, an infection caused by the bacterium *Orientia* *tsutsugamushi*, exhibit hypermetabolic generalized LAPs in FDG PET/CT images^12^. This condition, which affects approximately 1 million people annually, primarily in the Asia–Pacific region, can be fatal if not treated promptly^13–15^. Its symptoms, which are often under-recognized, necessitate early detection and effective treatment.

Systemic lupus erythematosus (SLE), a complex autoimmune disorder, also presents with LAP in its acute phase. This similarity to scrub typhus can lead to diagnostic confusion. SLE is characterized by diverse symptoms, ranging from mild cutaneous and constitutional symptoms to severe organ involvement^16^. LAP is a frequent, albeit nonspecific, manifestation in SLE patients, making careful assessment crucial for accurate diagnosis and for preventing the acceleration of disease activity^17,18^.

Despite extensive research efforts to distinguish among malignant LAPs, few studies have attempted to differentiate between various benign LAPs, including those caused by infections such as scrub typhus, and autoimmune diseases such as SLE. Accurate and early diagnosis is essential for initiating proper treatment, even in benign conditions, as it can significantly affect patient prognosis.

Given the clinical similarities between scrub typhus and SLE, particularly during acute phases, there is a clear need for early differential diagnosis. However, reliance on laboratory data results can delay diagnosis. This study was conducted with the expectation that FDG PET/CT imaging can be a valuable tool in differentiating between these two diseases, ultimately offering a faster and more effective approach to diagnosis.

## Results

### Patients and clinical data

Patients with scrub typhus were older than those with SLE (median age, 68.5 vs. 26.0 years, *P* = 0.017). The percentage of male patients was higher at 55.6% among all scrub typhus patients compared with 28.6% of SLE patients. However, there was no significant difference in the sex ratio between the two groups, which is likely due to the insufficient number of patients. The time interval from symptom onset to FDG PET/CT imaging was the same in the two groups, with a median of 7 days. Additionally, no significant differences were noted in terms of comorbidities such as diabetes mellitus or chronic obstructive pulmonary disease/asthma. With respect to clinical signs and symptoms, all scrub typhus patients presented with eschar, which is a pathognomonic sign of scrub typhus, while none of the SLE patients exhibited eschar or headache. Conversely, serositis was not observed in scrub typhus patients, whereas it was noted in four of seven SLE patients. All patients with scrub typhus and SLE presented with fever. No differences in other signs or symptoms were evident.

### Laboratory values

The albumin level of scrub typhus patients was greater than that of SLE patients (median 3.6 vs. 3.0 g/dL, *P* = 0.002). The normal albumin range at our institution is 3.5–5.2 g/dL. Although most albumin levels in scrub typhus patients were within the normal range, those measured in all SLE patients were below the normal range. The standard biochemical profile in lupus patients often includes a measurement of serum albumin, which can be low in these patients. This decline may be attributable to albumin catabolism induced by inflammation, proteinuria in individuals with lupus nephritis, or the extravasation of albumin due to increased capillary permeability^19,20^. The observed decrease in albumin levels in SLE patients aligns with the findings of previous research, confirming the utility of albumin as a marker of disease activity and implying that patients with SLE in this study were likely in an active stage at the time of FDG PET/CT examinations. The levels of aspartate aminotransferase (AST) and alanine aminotransferase (ALT) were also higher in scrub typhus patients than in SLE patients. The ALT level (median 86.0 vs. 19.0 IU/L) was significantly higher in scrub typhus patients (*P* = 0.012), while the AST level (median 91.5 vs. 38.0 IU/L) was higher with marginal significance (*P* = 0.055).

### Genotype of O. tsutsugamushi and systemic lupus Erythematosus disease activity index 2000

The genotype of *O*. *tsutsugamushi* was predominantly the Boryong strain in 16 cases, while 1 patient was determined to be infected with the Kawasaki strain. The genotype of the remaining case was not identified. Autoantibody profiles and Systemic Lupus Erythematosus Disease Activity Index 2000 (SLEDAI-2K) results were assessed in patients with SLE. In this cohort study, antinuclear antibody (ANA) positivity was observed in six of seven patients with SLE, with a notable mean high titer of 1:320. Subsequent analysis revealed a reduction in complement levels, encompassing C3 and C4, coupled with an increase in anti-double-stranded DNA (anti-dsDNA) antibody titers. Collectively, these findings provide compelling evidence of heightened disease activity in individuals with SLE. The mean SLEDAI-2K^21^ score, a clinical metric employed to evaluate SLE disease activity, was 13. These observed activity markers align with the previously mentioned clinical implication of hypoalbuminemia in the context of disease activity.

### Treatment

All scrub typhus patients received treatment with doxycycline, whereas SLE patients were given glucocorticoids and hydroxychloroquine. Additionally, mycophenolate mofetil was administered to six of seven SLE cases, while cyclophosphamide was given to the remaining SLE case. Patient characteristics and detailed values are presented in Table 1.

**Table 1:** Clinical characteristics and laboratory values of the patients with Scrub Typhus and those with SLE.

Characteristics	Scrub typhus (n = 18)		SLE (n = 7)		*P*-value
Demographic data, median (IQR)					
Age (years)	68.5	(58.3–75.8)	26.0	(23.0–63.5)	0.017*
Male, no. (%)	10	(55.6)	2	(28.6)	0.378
Time interval from symptom onset to FDG PET/CT imaging (days)	7.0	(4.3–8.0)	7.0	(4.5–17.0)	0.615
Comorbidities, no. (%)					
Diabetes mellitus	4	(22.2)	0	(0)	0.294
COPD/Asthma	1	(5.6)	0	(0)	1.000
Clinical signs & symptoms, no. (%)					
Headache	11	(61.1)	0	(0)	0.008*
Dyspepsia	7	(38.9)	5	(72.4)	0.202
Nausea/Vomiting	5	(27.8)	4	(57.1)	0.205
Abdominal pain	6	(33.3)	1	(14.2)	0.626
Fever	18	(100.0)	7	(100.0)	1.000
Rash	13	(72.2)	3	(42.8)	0.205
Eschar	18	(100.0)	0	(0)	0.000*
Serositis	0	(0)	3	(42.8)	0.015*
Nephritis	0	(0)	2	(28.5)	0.070
Laboratory values, median (IQR)					
WBC count, × 1000/m^3^	7.0	(4.7–10.3)	4.2	(3.3–5.2)	0.141
Lymphocyte count, × 1000/mm^3^	1.2	(0.8–1.6)	0.5	(0.3–1.1)	0.097
Platelet count, × 1000/mm^3^	128.5	(98.8–167.0)	221.0	(100.5–233.0)	0.244
PT, INR	1.1	(1.1–1.2)	1.0	(1.0–1.3)	0.534
Total bilirubin, mg/dL	0.61	(0.51–0.81)	0.38	(0.27–0.70)	0.097
Albumin, g/dL	3.6	(3.3–3.9)	3.0	(2.8–3.1)	0.002*
AST, IU/L	91.5	(76.0–139.0)	38.0	(22.5–90.5)	0.055
ALT, IU/L	86.0	(55.5–121.0)	19.0	(15.0–44.0)	0.012*
ALP, IU/L	105.5	(73.0–170.3)	129.0	(81.5–139.0)	0.836
Creatinine, mg/dL	0.8	(0.8–0.9)	0.7	(0.6–0.7)	0.097
hs-CRP, mg/dL	81.5	(59.6–145.4)	43.1	(41.0–87.0)	0.158
Genotype, no. (%)					
Boryong strain	16	(88.9)	Not tested		NA
Kawasaki strain	1	(0.1)	Not tested		NA
Not identified	1	(0.1)	Not tested		NA
Autoantibody profile, median (IQR)					
ANA positivity (%)	Not tested		6	(85.4)	NA
ANA titer	Not tested		1:320	(1:160–1:480)	NA
C3, mg/dL	Not tested		55.0	(33.5–60.0)	NA
C4, mg/dL	Not tested		10.0	(7.2–17.0)	NA
Anti-dsDNA antibody positivity (%)	Not tested		5	(71.4)	NA
Anti-dsDNA antibody titer, IU/mL	Not tested		20.7	(9.0–200.0)	NA
Disease activity score, median (IQR)					
SLEDAI-2 K	Not tested		13.0	(10.5—14.5)	NA
Steroid use from 3 months prior to the time of FDG PET/CT, no. (dose)	0 (0)		1	(2.5 mg/day of PD equivalent)	NA
Treatment, no. (%)					
Doxycycline	18	(100.0)	Not administered		NA
Glucocorticoid	Not administered		7	(100.0)	NA
Hydroxychloroquine	Not administered		7	(100.0)	NA
Mycophenolate mofetil	Not administered		6	(85.7)	NA
Cyclophosphamide	Not administered		1	(14.7)	NA

Data are presented as median (interquartile range) or number (percentage). Abbreviations: ALP, alkaline phosphatase; ALT, alanine aminotransferase; ANA, anti-nuclear antibody; anti-dsDNA antibody, anti-double stranded DNA antibody; AST, aspartate aminotransferase; C3, Complement 3; C4, Complement 4; COPD, chronic obstructive pulmonary disease; FDG, fluorodeoxyglucose; hs-CRP, high-sensitivity C-reactive protein; IQR, interquartile range; NA, Not applicable; PD, Prednisolonone; PET/CT, Positron Emission Tomography/Computed Tomography; PT, Prothrombin time; SLE, systemic lupus erythematosus; SLEDAI-2 K, systemic lupus erythematosus disease activity index 2000; WBC, white blood cell. *P-value < 0.05.

### F-18 FDG PET/CT parameters

**Figure 1:**
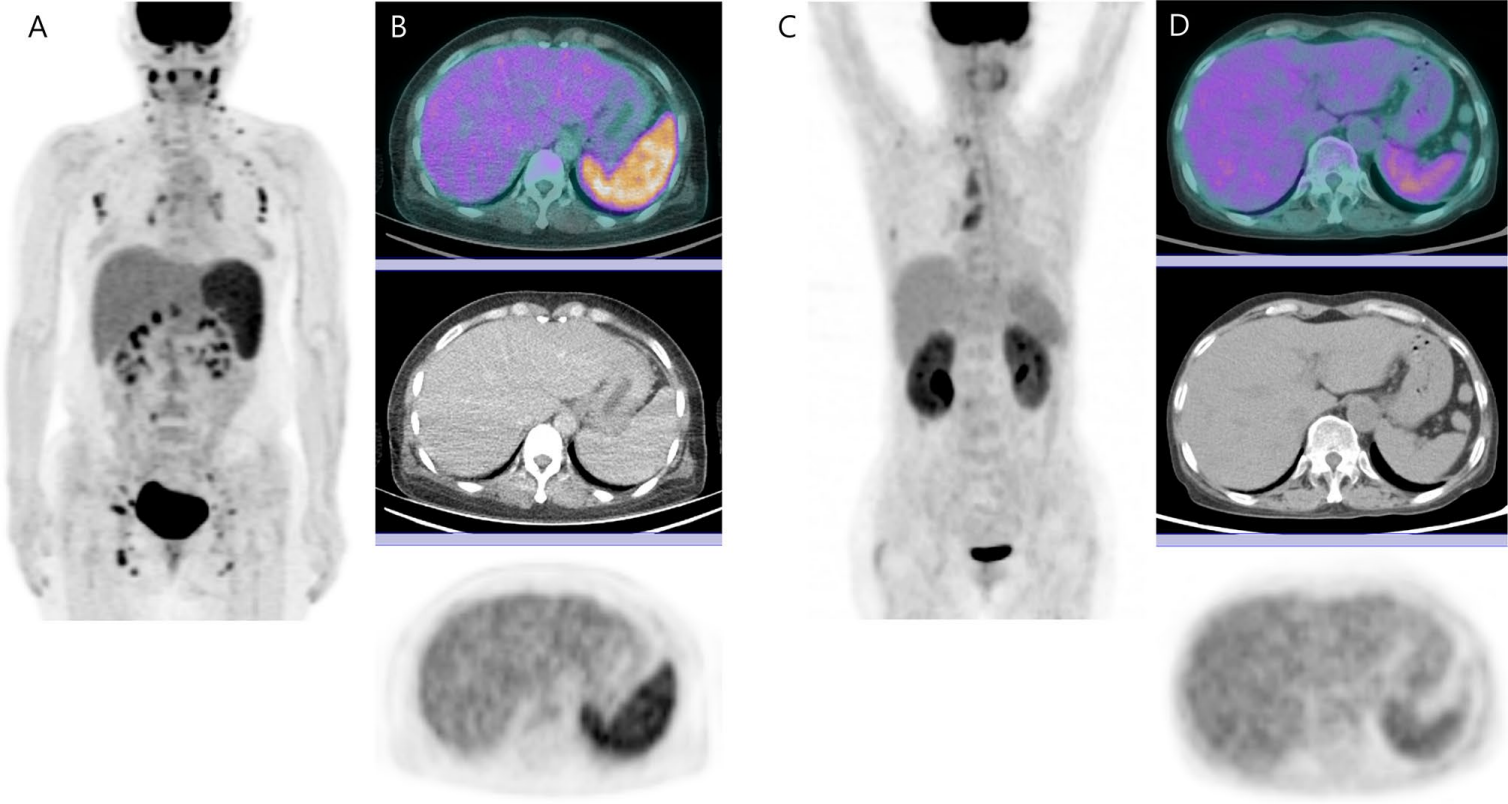
FDG PET/CT images of a 49 year-old female patient with scrub typhus and a 63 year-old female patient with SLE. The patient with scrub typhus demonstrates extensive involvement of lymph nodes from both cervical to inguinal areas (**A**), whereas the patient with SLE shows lymph nodes in the right supraclavicular and mediastinal areas (**C**). FDG uptake levels in the lymph nodes, spleen, and liver are higher in the patient with scrub typhus (**B**) compared to those in the patient with SLE (**D**).

An analysis of lengths found no statistical differences in the maximal short axis of the involved lymph node, the maximal length of the spleen, or the maximal anterior–posterior length of the liver between patients with scrub typhus and those with SLE. However, the D_max_ of scrub typhus patients was longer at 72.2 cm compared with 57.5 cm in SLE patients, indicating that LAP is more generalized in scrub typhus patients compared with those with SLE. The D_max_ of scrub typhus patients was ≥ 70 cm in 15 of 18 cases, while, only one of the seven SLE patients showed a D_max_ > 70 cm. In a comparison of metabolic parameters, the SUV_max_ of the lymph nodes, spleen, and liver was significantly higher in patients with scrub typhus, while the SUV_mean_ for the liver and spleen did not differ between the two groups. Various FDG PET/CT parameters are listed in Table 2, and Fig. 1 depicts typical cases of acute-phase scrub typhus and SLE.

**Table 2:** FDG PET/CT parameters in the patients with Scrub Typhus and those with SLE.

Parameters	Scrub typhus median (IQR)		SLE median (IQR)		*P*-value
Maximal short axis of lymph node (cm)	1.10	(0.93–1.20)	1.40	(1.15–1.60)	0.064
Maximal length of spleen (cm)	11.05	(10.18–12.63)	10.90	(8.85–13.05)	0.657
Maximal A-P length of liver (cm)	15.55	(14.70–16.18)	14.70	(13.85–15.15)	0.097
D_max_ (cm)	72.20	(70.35–76.50)	57.50	(40.15–66.45)	0.002*
SUV_max_ of lymph node	10.51	(8.05–14.57)	5.35	(5.32–8.42)	0.005*
SUV_max_ of spleen	5.94	(4.72–6.32)	3.23	(3.01–5.34)	0.009*
SUV_max_ of liver	3.70	(3.33–4.20)	2.99	(2.61–3.21)	0.003*
SUV_mean_ of spleen	3.63	(3.03–4.20)	2.33	(2.12–3.81)	0.220
SUV_mean_ of liver	2.20	(1.91–2.44)	1.81	(1.76–2.00)	0.141

Data are presented as median (interquartile range). Abbreviation: A-P, anterior posterior; FDG, fluorodeoxyglucose; IQR, interquartile range; PET/CT, Positron Emission Tomography/Computed Tomography; SLE, systemic lupus erythematosus. *P-value < 0.05.

## Discussion

This study investigated FDG PET/CT findings in patients with scrub typhus and SLE, an area largely covered by case reports with limited patient samples^22,23^. Our research incorporated a larger cohort of patients in the acute phase of the disease, allowing for a more robust comparison between scrub typhus and SLE based on FDG PET/CT imaging.

An important aspect of this investigation is the identification of distinct discriminative features using FDG PET/CT, particularly between infectious and inflammatory diseases, which often exhibit overlapping characteristics. We found that measurement of the sizes of the lymph node, spleen, and liver via CT does not significantly aid in differentiating between these two conditions. In contrast, the use of SUV_max_ in FDG PET imaging proved to be a pivotal metric for differentiation, due to its minimal interobserver and intraobserver variability, making it a practical and reliable option in clinical settings.

Another innovative aspect of this study is the application of D_max_ to a non-lymphoma patient group, where it is traditionally an independent prognostic factor in lymphoma^24–26^. This research demonstrates D_max_'s potential as a valuable parameter in distinguishing between scrub typhus and SLE. Despite D_max_ focusing on anatomical data, the precision in identifying involved lymph nodes, primarily through FDG-avidity, highlights the importance of FDG PET imaging.

Scrub typhus and SLE share common clinical features, such as fever, LAP, and thrombocytopenia, which complicates attempts to distinguish between them, particularly in ANA-negative SLE cases or those involving scrub typhus with undetected eschars. Criteria established in 2019 by the European Alliance of Associations for Rheumatology and American College of Rheumatology advise against attributing a symptom to SLE if there is a more likely alternative explanation. Accurate and prompt diagnosis is crucial because distinct management approaches are required for both diseases. This study highlights the clinical significance of FDG PET/CT findings as a tool for differentiating between the two conditions, contributing valuable insights to clinical practice.

The present study also addresses the variability in eschar manifestation in scrub typhus patients across geographic regions^27–30^. This variation presents a diagnostic challenge, particularly in areas with low rate of eschar prevalence. For example, East Asia reports the highest eschar occurrence (78.7%), with noticeably lower rates in Oceania, Southeast Asia, and South Asia^31^. Such discrepancies necessitate the use of alternative diagnostic methods beyond relying solely on the presence of eschar, to avoid misdiagnosis or delays in diagnosis, particularly in regions where eschar appearances are less frequent.

Although no cases were observed in our study where steroids could affect FDG PET/CT images, special attention is required during FDG PET/CT imaging because steroids can influence lymph node size and FDG uptake in various organs. Glucose transporter and hexokinase play crucial roles in the process of FDG uptake into cells. Numerous studies have reported that steroids not only increase blood glucose levels but inhibit the actions of glucose transporter and hexokinase^32–34^. When steroids are administered, various FDG PET/CT parameters, including SUV_max_, are likely to be measured at lower levels.

This study has several limitations. First, its retrospective nature led to variation in demographics and comorbidities between groups, although the uniformity in the time interval from symptom onset to imaging lends credibility to the FDG PET/CT analysis. Our small patient sample of only 18 scrub typhus and 7 SLE patients limits the statistical power of certain findings, such as sex distribution and AST levels. Further research with larger cohorts is necessary to confirm these findings.

In the future, we intend to conduct research that differs from many previous studies that focused on distinguishing malignant from benign LAP. Instead, we aim to elucidate more disease-specific findings using various FDG PET/CT parameters regardless of whether the disease is malignant or benign, infectious, or autoimmune. For these studies to be meaningful, they should be conducted in large cohorts at multiple centers with prospective methods.

While it does not differentiate among all types of LAPs, this study takes a significant step towards simplifying the diagnosis of various LAPs by providing a tool to distinguish between scrub typhus and SLE. Our findings suggest that FDG PET/CT parameters can minimize unnecessary tests and facilitate timely and appropriate diagnostic interventions for these conditions.

## Methods

### Patients and data collection

From August 2006 to July 2023, a total of 18 patients diagnosed with scrub typhus and seven diagnosed with SLE underwent FDG PET/CT imaging before treatment. Scrub typhus was confirmed by (1) an increase in the indirect immunofluorescence assay (IFA) immunoglobulin M titer ≥ 1:160 against *O*. *tsutsugamushi*, (2) an increase in the IFA immunoglobulin G titer against *O*. *tsutsugamushi* to ≥ 1:256, (3) a ≥ four-fold increase in the IFA titer against *O*. *tsutsugamushi*, or (4) the presence of a positive reaction in a nested polymerase chain reaction (PCR) targeting the 56-kDa gene of *O*. *tsutsugamushi*. Clinical characteristics including age, sex, comorbidities, signs and symptoms, and laboratory values were collected from electronic medical records. We examined data from individuals diagnosed with SLE who were ≥ 18 years of age and who were categorized using either the Systemic Lupus International Collaborating Clinics classification criteria for SLE or the 2019 European Alliance of Associations for Rheumatology/American College of Rheumatology criteria^35,36^. Patients with SLE who had concurrent diagnoses of infection or malignancy were excluded from the analysis.

### Genotyping by DNA amplification and sequencing

Peripheral blood mononuclear cells collected from acute-phase blood samples of scrub typhus patients were purified using a QIAamp DNA blood mini kit (QIAGEN GmbH, Hilden, Germany) according to the manufacturer’s protocol. A nested PCR was performed as described previously by Kim et al^37^. Primers 34 (forward, 5′-TCA AGC TTA TTG CTA GTG CAA TGT CTGC-3′; the 56-kDa gene based on the Gilliam strain) and 55 (5′-AGG GAT CCC TGC TGC TGT GCT TGC TGCG-3′) were used in the first PCR amplification. Nested PCR primers 10 (5-GAT CAA GCT TCC TCA GCC TAC TAT AAT GCC-3) and 11 (5-CTA GGG ATC CCG ACA GAT GCA CTA TTA GGC-3) were used in the second PCR amplification to generate a fragment of 483 base pairs. The amplified PCR products were confirmed with 1.2% agarose gel electrophoresis, purified using a QIA quick gel extraction kit (QIAGEN) and sent to COSMO Genetech (Seoul, Korea) for sequencing.

### F-18 FDG PET/CT acquisition

All patients fasted for ≥ 6 h before intravenous injection of F-18 FDG. The blood glucose levels of all patients were ≤ 140 mg/dL^38^. Approximately 5.18 MBq (0.14 mCi) of F-18 FDG per kilogram of body weight was administered intravenously. After 60 min, images were obtained from the base of the skull to the proximal thigh using either a Biograph 40 TruePoint PET/CT scanner or Biograph 16 PET/CT scanner (Siemens Medical Solutions, Knoxville, TN, USA). A CT scan was performed using a continuous spiral method (120 kV, 160 mA, 0.5 s rotation time). Subsequently, a PET scan was conducted using a three-dimensional mode for 2 min per bed position. The acquired PET data underwent iterative reconstruction employing an ordered-subset expectation maximization algorithm (128 × 128 matrix, 3.27-mm slice thickness, subset: 21, iterations: 2).

### Image interpretation

**Figure 2:**
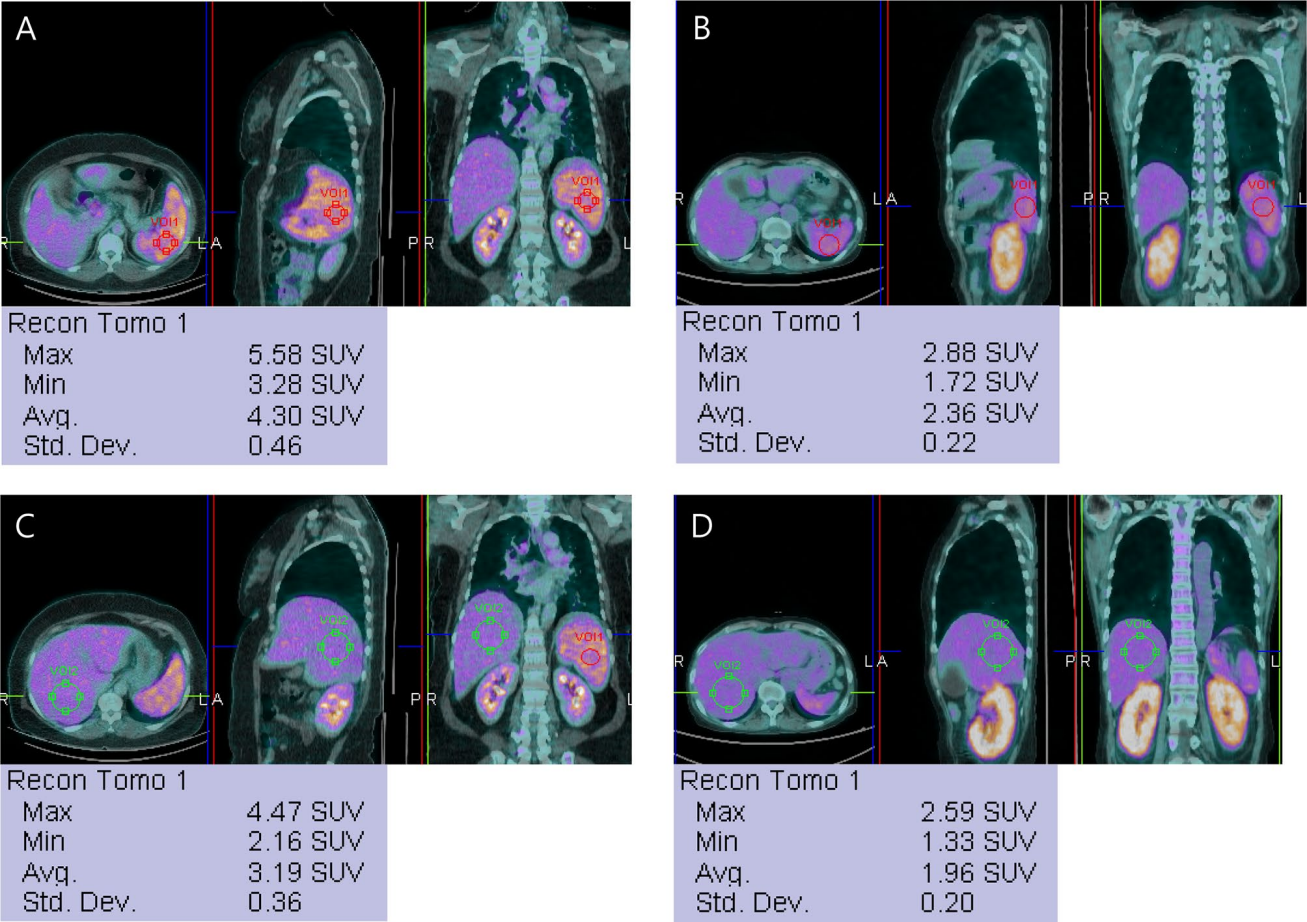
A spherical volume of interest (VOI) 3 cm in diameter is placed on the posterior portion of the spleen. The patient with scrub typhus (**A**) shows higher SUV_max_ and SUV_mean_ than the patient with SLE (**B**). The SUV_mean_ of the liver is also measured using a VOI 5 cm in diameter. Like the metabolic parameters in the spleen, the liver also shows higher glucose metabolism in the patient with scrub typhus (**C**) than in the patient with SLE (**D**).

We reviewed the FDG PET/CT images on a workstation (Syngo MI applications, Flexible Display 7.0.7.7; Siemens Medical Solutions, Erlangen, Germany) and measured the maximal short axis of the involved lymph node, the maximal length of the spleen, and the maximal anterior–posterior length of the liver on the transverse CT plane of FDG PET/CT. We then measured the distance between the two lesions that were farthest apart (D_max_) on the coronal CT plane^39^. We measured the metabolic parameters of the maximum standardized uptake value (SUV_max_) of the lymph node, spleen, and liver and the mean standardized uptake value (SUV_mean_) of the liver and spleen. To measure SUV_max_, a volume of interest (VOI) was carefully drawn to be slightly larger than the target region in the axial, coronal, and sagittal planes. The SUV_mean_ was measured using VOIs 3 cm and 5 cm in diameter in the posterior portion of the spleen and the liver, respectively (Fig. 2). SUV_max_, which was defined as the maximum SUV within the VOI, was calculated as the concentration of highest tumor activity in the VOI (MBq/mL) × total body weight (kg)/injected radioactivity (g/MBq). The SUV_mean_ value, representing the average metabolism, was calculated as the summed SUV divided by the number of voxels within the VOI.

### Statistical analysis

Statistical analysis was conducted using SPSS software (version 23, IBM Corporation, Armonk, NY, USA), and a *P* value < 0.05 was considered statistically significant. Two groups were compared using Fisher's exact test for sex, comorbidities, and signs and symptoms, while continuous values were compared using the Mann–Whitney *U* test. A comparison of various parameters in FDG PET/CT images was also performed using the Mann–Whitney *U* test, which is a non-parametric version of the independent *t*-test.

### Ethical approval

This study was conducted in accordance with Good Clinical Practice Guidelines and the Declaration of Helsinki. It was approved by the Institutional Review Board (IRB) of Jeonbuk National University Hospital (IRB registration no. 2023-09-048), and the need for informed consent was waived because of the retrospective nature of this study.

## Data Availability

The datasets generated and/or analysed during the current study are available in the NCBI repository [Accession number to datasets: KY946045, KY946102, KY946106, KY946100, KY946062, KY946099, KY946043, KY946101, KY946009, MK613928, KC424618, KY957849, KY946004, AB751260, MG844361, KY946036, KT957856, KY946004].
